# Supplementary material for: Direct Comparison of Two Different Definitions with Biochemical Recurrence after Low-Dose-Rate Brachytherapy for Prostate Cancer
Source: Curr Oncol. 2023 Feb 26;30(3):2792–800. doi: 10.3390/curroncol30030212 (PMC10047870; doi:10.3390/curroncol30030212)
Supplement: Supplementary file 1 [file curroncol-30-00212-s001.zip › curroncol-2178310-supplementary.pdf]

S1.

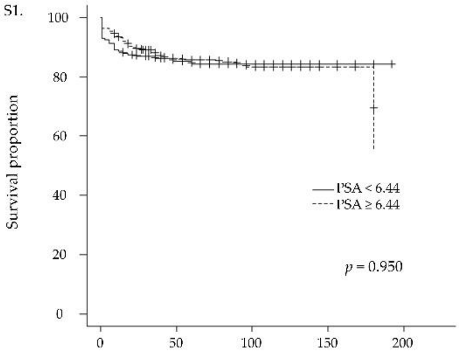

Number at risk	0	50	100	150	200
PSA < 6.44	238	183	88	20	0
PSA ≥ 6.44	238	172	85	14	0

S2.

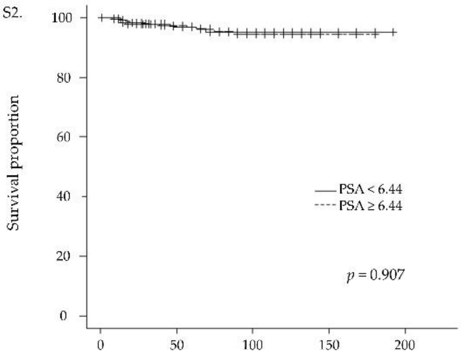

Number at risk	0	50	100	150	200
PSA < 6.44	238	194	91	21	0
PSA ≥ 6.44	238	181	85	14	0

S3.

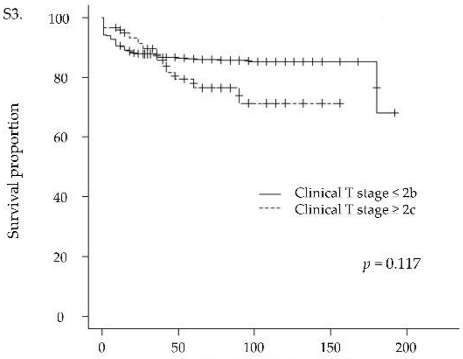

Number at risk	0	50	100	150	200
Clinical T stage ≤ 2b	417	325	163	33	0
Clinical T stage ≥ 2c	59	30	10	1	0

S4.

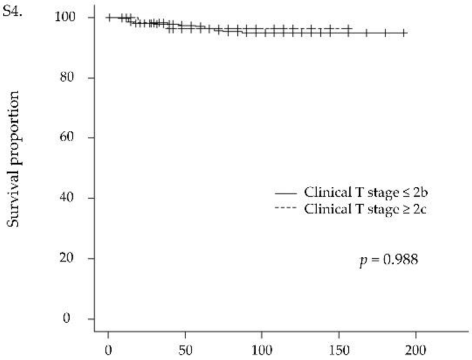

Number at risk	0	50	100	150	200
Clinical T stage ≤ 2b	417	344	166	34	0
Clinical T stage ≥ 2c	59	31	10	1	0

S5.

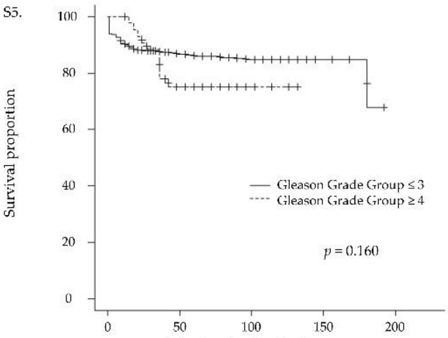

Number at risk	0	50	100	150	200
Gleason Grade Group ≤ 3	432	332	165	34	0
Gleason Grade Group ≥ 4	44	23	8	0	0

S6.

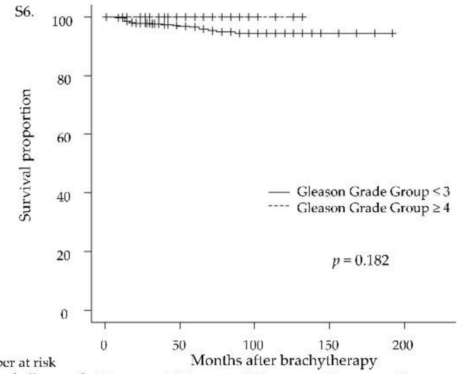

Number at risk	0	50	100	150	200
Gleason Grade Group ≤ 3	432	349	168	35	0
Gleason Grade Group ≥ 4	44	26	8	0	0

S7.

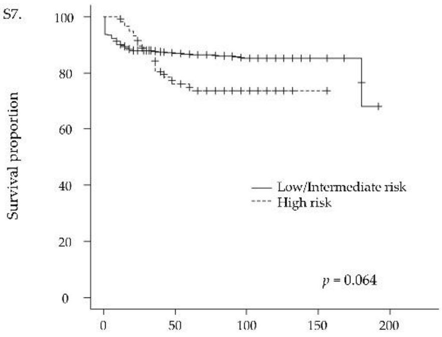

Number at risk	0	50	100	150	200
Low/Intermediate risk	417	324	161	33	0
High risk	59	31	12	1	0

S8.

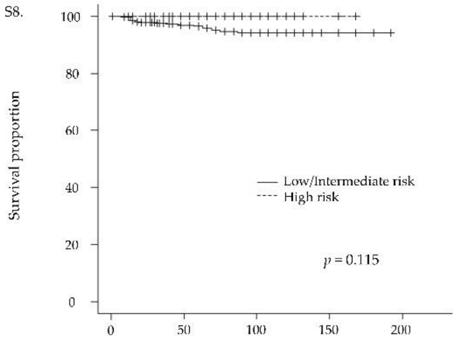

Number at risk	0	50	100	150	200
Low/Intermediate risk	417	340	163	33	0
High risk	59	35	13	2	0

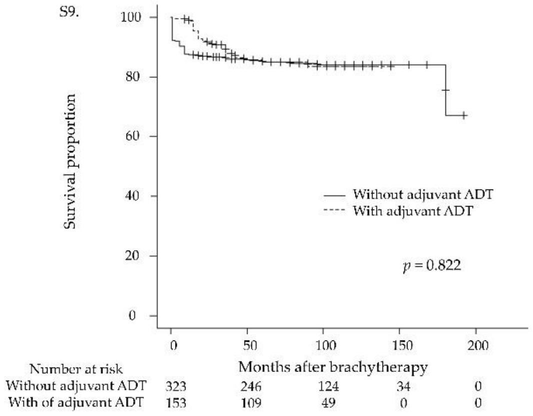

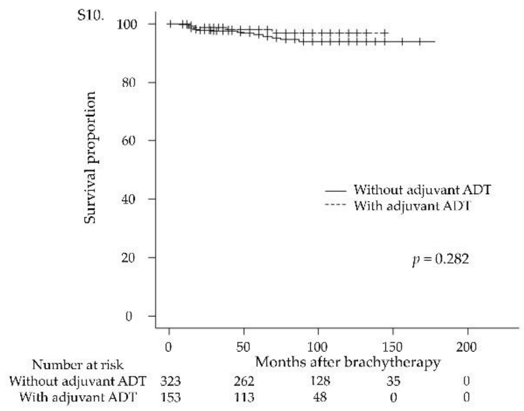

Figure S1-10. Kaplan-Meier estimates of biochemical recurrence-free survival (BRFS) for patients with PCa receiving low-dose-rate brachytherapy were calculated using the definition of biochemical recurrence after radical prostatectomy (RP) and the definition given in the Japanese Prostate Cancer Outcomes Study (J-POPS) with respect to pretreatment clinical covariates, including initial prostate-specific antigen (PSA) level, clinical T stage, biopsy Gleason Grade Group (bGG), National Comprehensive Cancer Network (NCCN) risk classification, and adjuvant androgen deprivation therapy (ADT). (S1) With the RP definition, the 5-year BRFS rates were 84.2% in the group PSA < 6.44 ng/mL and 85.8% in those PSA ≥ 6.44 ng/mL. (S2) According to the J-POPS definition, the 5-year BRFS rates in the groups PSA < 6.44 ng/mL and ≥ 6.44 ng/mL were 96.9% and 96.8%, respectively. (S3) Based on the RP definition, the 5-year BRFS rates were 86.0% in the group clinical T stage ≤ 2b and 76.4% in those clinical T stage ≥ 2c. (S4) Regarding to the J-POPS definition, the 5-year BRFS rates in the groups clinical T stage ≤ 2b and clinical T stage ≥ 2c were 97.0% and 96.2%, respectively. (S5) With the RP definition, the 5-year BRFS rates were 85.9% in patients with bGG ≤ 3 and 75.0% in patients with bGG ≥ 4. (S6) According to the J-POPS definition, the 5-year BRFS rates in patients with bGG ≤ 3 and bGG ≥ 4 were 96.6% and 100%, respectively. (S7) Based on the RP definition, the 5-year BRFS rates were 86.4% in the group low/intermediate risk and 73.6% in those high risk. (S8) Regarding to the J-POPS definition, the 5-year BRFS rates in the groups low/intermediate risk and high risk were 100% and 96.5%, respectively. (S9) With the RP definition, the 5-year BRFS rates were 85.0% in the group without adjuvant ADT and 84.9% in those with adjuvant ADT. (S10) According to the J-POPS definition, the 5-year BRFS rates in the groups without adjuvant ADT and with adjuvant ADT were 96.4% and 97.9%, respectively.
